# NMR spectroscopy enables simultaneous quantification of carbohydrates for diagnosis of intestinal and gastric permeability

**DOI:** 10.1038/s41598-018-33104-8

**Published:** 2018-10-02

**Authors:** Sarah Stryeck, Angela Horvath, Bettina Leber, Vanessa Stadlbauer, Tobias Madl

**Affiliations:** 1 Gottfried Schatz Research Center for Cell Signaling, Metabolism and Aging, Molecular Biology and Biochemistry, Medical University of Graz, 8010 Graz, Austria; 2 Department of Internal Medicine, Division of Gastroenterology and Hepatology, Medical University of Graz, 8010 Graz, Austria; 3 Center for Biomarker Research in Medicine (CBmed), 8010 Graz, Austria; 4 Department of Surgery, Division of Transplantation Surgery, Medical University of Graz, 8010 Graz, Austria; 5 BioTechMed/Graz, Graz, Austria

## Abstract

Increased intestinal or gastric permeability is one of the major hallmarks of liver cirrhosis. The current gold standard for diagnosis of aberrant gut permeability due to disease is the triple-sugar test, where carbohydrates are orally administered and urinary excretion is measured. Hereby, elevated lactulose levels indicate intestinal permeability, whereas increased sucrose levels reveal gastric permeability. However, reliable detection and quantification of these sugars in a complex biological fluid still remains challenging due to interfering substances. Here we used Nuclear Magnetic Resonance (NMR) spectroscopy with a simple and fast protocol, without any additional sample extraction steps, for straight-forward simultaneous quantification of sugars in urine in order to detect increased intestinal and gastric permeability. Collected urine samples were diluted in buffer and one- and two-dimensional proton spectra were recorded in order to reveal carbohydrate concentrations in individual urine samples containing mannitol, sucrose and/or lactulose. Overall, this article presents a fast and robust method for simultaneous quantification of different sugars down to low micro-molar concentrations for research studies and can be further extended for clinical studies with automation of the quantification process.

## Introduction

Liver cirrhosis is a major global health burden with a continuously rising incidence. Besides common disease indications like altered liver histology, alterations in hepatic venous pressure or blood parameters, increased intestinal and gastric permeability are hallmarks of advanced liver disease resulting in severe septic complications. Therefore analysis of an increased intestinal permeability is inevitable for preventing profound infections^1^.

Intestinal and upper digestive tract permeability is functionally analyzed by orally administering chemically inert, not metabolized reference substances to patients and by tracing their distribution and finally renal excretion. Differences in cellular pathways of these passively absorbed sugars, make the determination of deficiencies of mucosal barriers, and absorbance efficiency in the gut feasible. The current gold standard is a combination of the carbohydrates mannitol, lactulose, and sucrose^2–5^. Hereby, mannitol uses a transcellular pathway, whereas lactulose and sucrose are transported paracellularly through intercellular junctions between enterocytes. The disaccharide sucrose is, after passing the first part of the digestive tract, cleaved into glucose and fructose in the jejunum. Therefore, intact sucrose molecules serve as a marker for gastric permeability^6^. For the ‘triple-sugar test’ elevated lactulose or sucrose levels in urine are a common readout for increased intestinal or gastric permeability, respectively, due to a huge increase in paracellular transport of metabolites into the systemic circulation and subsequent urinary excretion. Mannitol serves as a reference compound for sugar intake, which is always excreted via urine in healthy individuals. However, decreased mannitol levels in urine reveal deficiency in mucosal absorbance caused by villus shortening^7,8^. Sugar-dependent enzymatic assay and chromatographic methods are commonly used in order to detect and quantify these compounds^8,9^. However, these approaches are limited in terms of their accuracy, throughput, and interference with other compounds often present in human urine, such as interference of other carbohydrates such as galactose in enzymatic assays^7,10^. For instance, HPLC/mass spectrometry (MS)-based sugar quantification requires sample preparation steps in order to eliminate compounds causing signal suppression in tandem MS detectors, such as sodium ions. Sample preparation steps are not required in Nuclear Magnetic Resonance (NMR) spectroscopy-based measurements; hereby urine samples can be measured directly. Both NMR and MS can be fully quantitative, providing that guidelines for sample preparation and experimental setup are followed^9,11–16^. One potential drawback of NMR spectroscopy compared to MS-based approaches is the relatively low sensitivity. However, this limitation is only relevant in detection of low concentrated compounds. Since carbohydrate concentrations in urine are in the µM range, they can be detected by standard NMR experiments with 30–60 minutes measurement time^17^. A pathologically increased intestinal permeability index (lactulose/mannitol ratio >0.07) would presume even higher concentrations of carbohydrates, which require only short measurement times of less than 10 minutes^3,4^.

NMR spectroscopy is well-applicable for quantification of metabolites in complex mixtures^5^. A first approach to determine intestinal permeability by quantification of lactulose and mannitol levels in urine has been proposed by Jayalakshmi *et al*. and, more recently, by Kumar *et al*.^18,19^. However, the standard 1D NMR experiments used in this approach suffer from severe signal overlap in the sugar NMR spectral region. Here, we propose an alternative to overcome these drawbacks and to detect not only intestinal, but also gastric permeability by simultaneous sucrose detection. By using ^1^H homo-nuclear J-resolved 2D correlation experiments (JRES) overlap of NMR signals is largely reduced, resulting in a reliable and highly reproducible identification and quantification of metabolites^20,21^. In contrast to conventional 1D NMR experiments conducted on metabolite samples, these NMR experiments introduce an additional dimension separating the chemical shift in the first dimension from the scalar (J)-coupling in the second dimension. Projection of the 2D spectrum yields a decoupled spectrum in which the multiplets are reduced to single peaks and signal overlap is resolved^20,22^. Aside from the benefits, 2D JRES experiments bring along several features requiring prudent usage of these experiments. These drawbacks include longer acquisition times, higher technical variability and phase-twisted line shapes resulting in quantification errors. However, with our study we show that advanced knowledge on pros and cons of this NMR-based metabolomics tool enables the adequate usage for particular research questions^20,23,24^. Our study presents a rapid, robust, and non-invasive NMR-based ‘triple-sugar test’ which enables simultaneous quantification of sugars in human urine using NMR spectroscopy in order to determine gastric and/or intestinal permeability in samples for research studies, but also for larger sample sizes in clinical applications. In addition to sugar quantification, untargeted urinary metabolite profiles are obtained for free without the need for any additional measurement time.

## Results

### NMR signals of sugars can be well separated in urine

**Figure 1:**
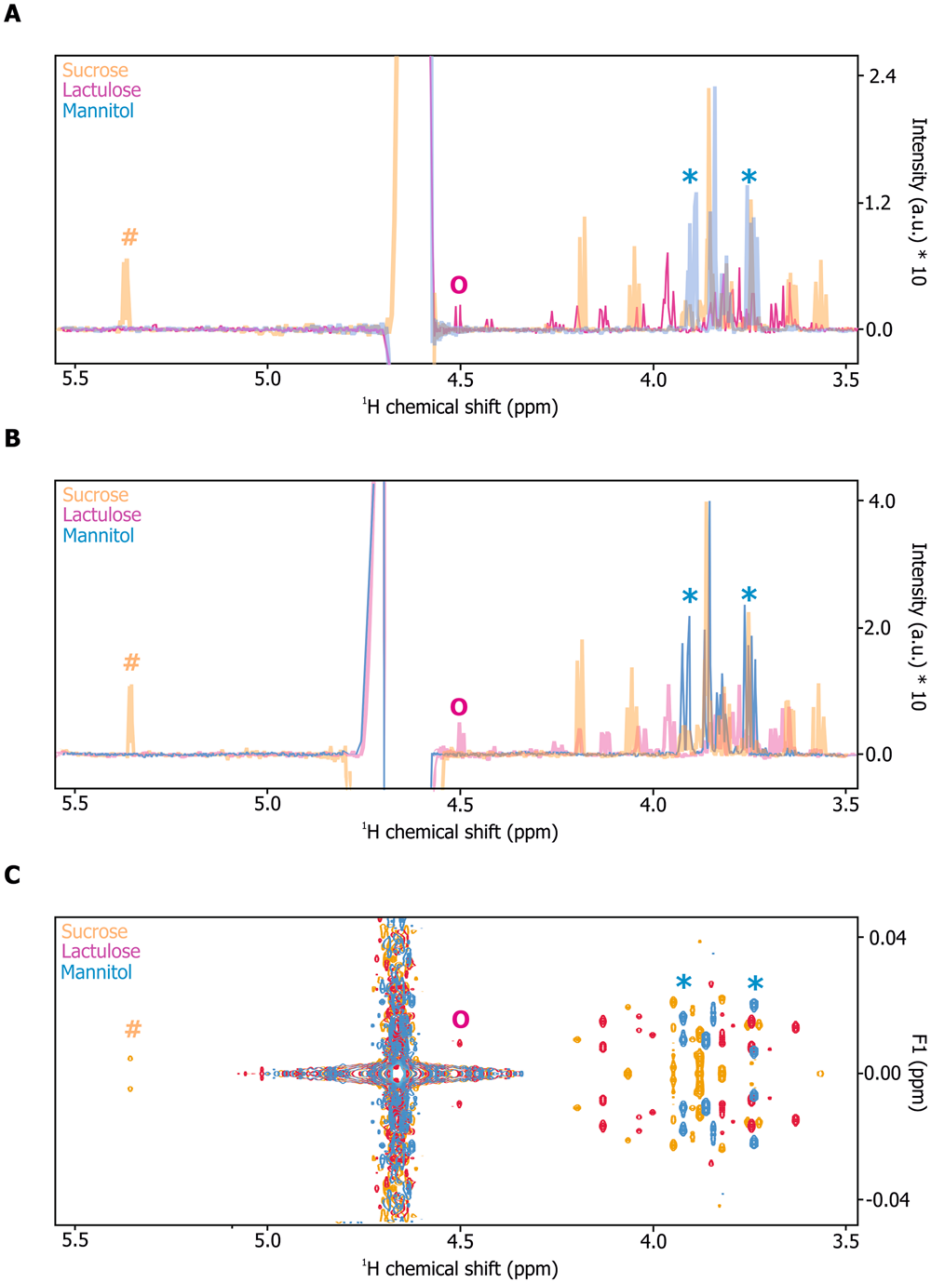
(**A–C**) Reference NMR spectra of carbohydrate standards (sugar region). (**A**) Overlay of ^1^H 1D CPMG experiments of sucrose (orange), lactulose (magenta) and mannitol (light blue) (each 100 µM). (**B**) Overlay of ^1^H 1D NOESY experiments of sucrose (orange), lactulose (magenta) and mannitol (light blue) (each 100 µM). (**C**) Overlay of ^1^H 2D J-resolved experiments of sucrose (orange), lactulose (magenta) and mannitol (light blue) (each 100 µM). Characteristic carbohydrate signals were marked by orange hash (sucrose), magenta circle (lactulose) or light blue asterisks (mannitol).

**Figure 2:**
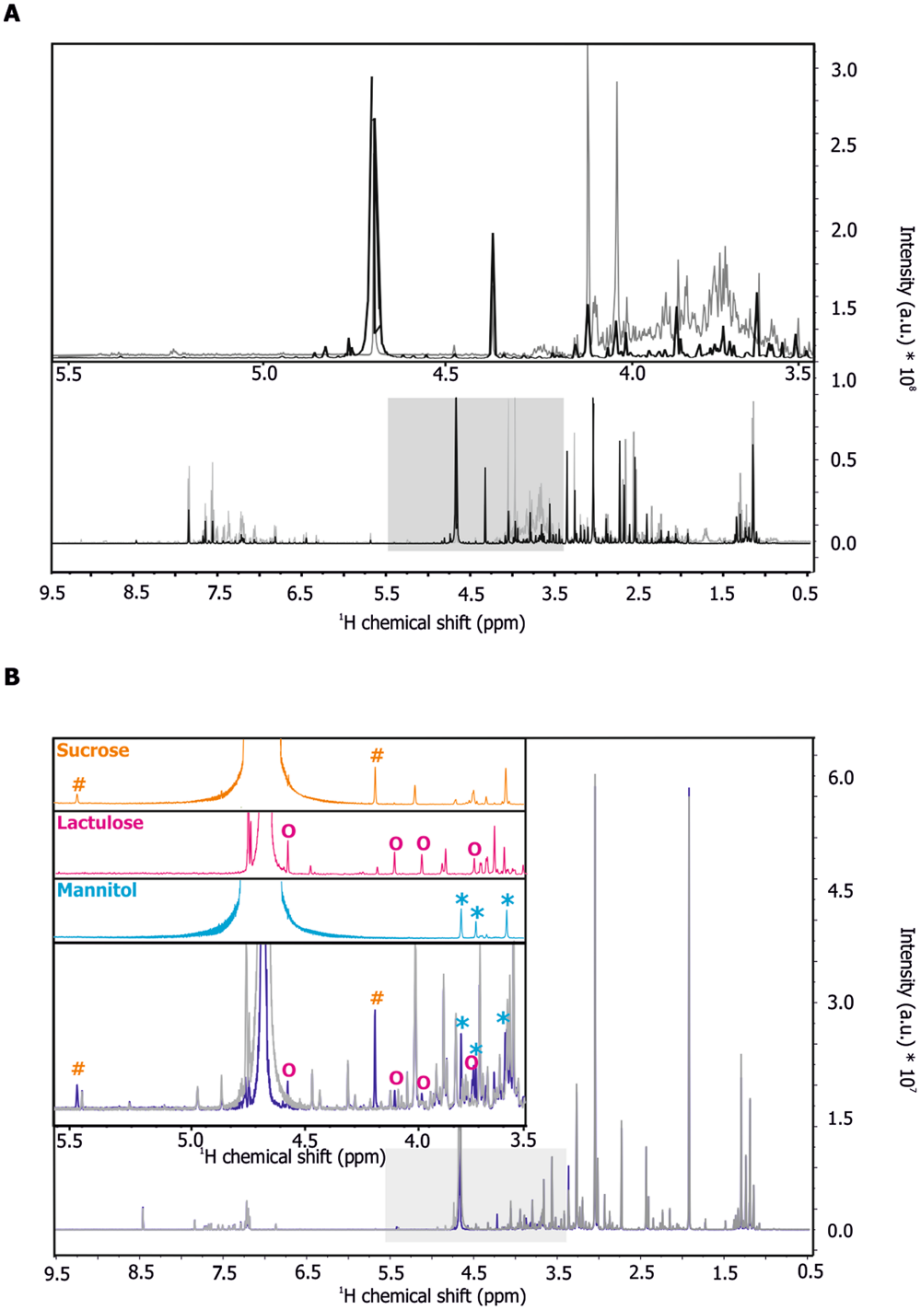
(**A,B**) NMR spectroscopy of urine samples. (**A**) Overlay of a ^1^H 1D CPMG experiment (grey) and the projection of a ^1^H J-resolved 2D experiment (black) of a urine sample after sugar intake. Both experiments show NMR signals of all metabolites harboring non exchangeable protons. By resolving the J-coupling in the indirect dimension, the spectral signatures are simplified. (**B**) Projections of ^1^H J-resolved 2D experiments of reference compounds sucrose (orange), lactulose (magenta), and mannitol (light blue) (each 100 µM). Corresponding resonances of these sugars in a human urine sample before (grey) and after sugar intake (marine blue) are indicated with orange hash (sucrose), magenta circle (lactulose) or light blue asterisks (mannitol).

Proton/^1^H-detected NMR experiments record resonances of all protons present in small molecules resulting in a NMR signal for chemically equivalent protons. Most metabolites, including mannitol, sucrose and lactulose, comprise several chemically different protons leading to complex spectral patterns, signal overlap, and this complicates reliable identification and quantification of metabolites (Fig. 1A and B; for full ^1^H NMR spectra see Fig. S1A and B). NMR spectroscopy offers several options enabling the simplification of highly overlapped NMR spectra through (effective) homo-nuclear decoupling. Broadband homo-nuclear decoupled experiments, also known as ^1^H pure shift experiments, are well-suited for concentrated samples, but suffer from low sensitivity, which makes long measurement times necessary (Suppl. Fig. 2). Therefore, we recorded ^1^H homo-nuclear J-resolved 2D correlation experiments in order to reduce the complexity of NMR spectra with moderate measurement times. Using this pulse sequence, scalar coupling of neighboring protons, and therefore splitting of NMR signals can be resolved in the ordinate^22^ (Fig. 1C). Projection of these 2D spectra results in one-dimensional data showing NMR signals for each metabolite and lacking the complex splitting pattern observed in conventional 1D NMR experiments (Fig. 2A).

To ensure reliable identification and quantification of sugars, NMR reference spectra were recorded for the isolated compounds, for urine samples containing sugars before and after sugar intake (Fig. 2B) and spiked with the corresponding carbohydrates (Suppl. Fig. 3). In addition, two-dimensional homonuclear ^1^H-^1^H TOCSY experiments were used in order to verify the assignment of the peaks in the 1D spectrum (Suppl. Fig. 4A–C).

### Quantification of sugars in urine

Integrals of NMR signals in ^1^H homo-nuclear J-resolved 2D correlation experiments can provide quantitative information for the compound of interest given the relaxation time of the compounds is comparable, or an appropriate relaxation delay is used (Suppl. Fig. 5). However, even with short relaxation delays, correct information on concentrations can be obtained providing that reference spectra recorded with the same setup are used as external reference for quantification. Here we recorded reference spectra of all sugars and including TSP and used these reference spectra as input to fit and determine the sugar concentrations in Chenomx (see Methods section for details).

**Figure 3:**
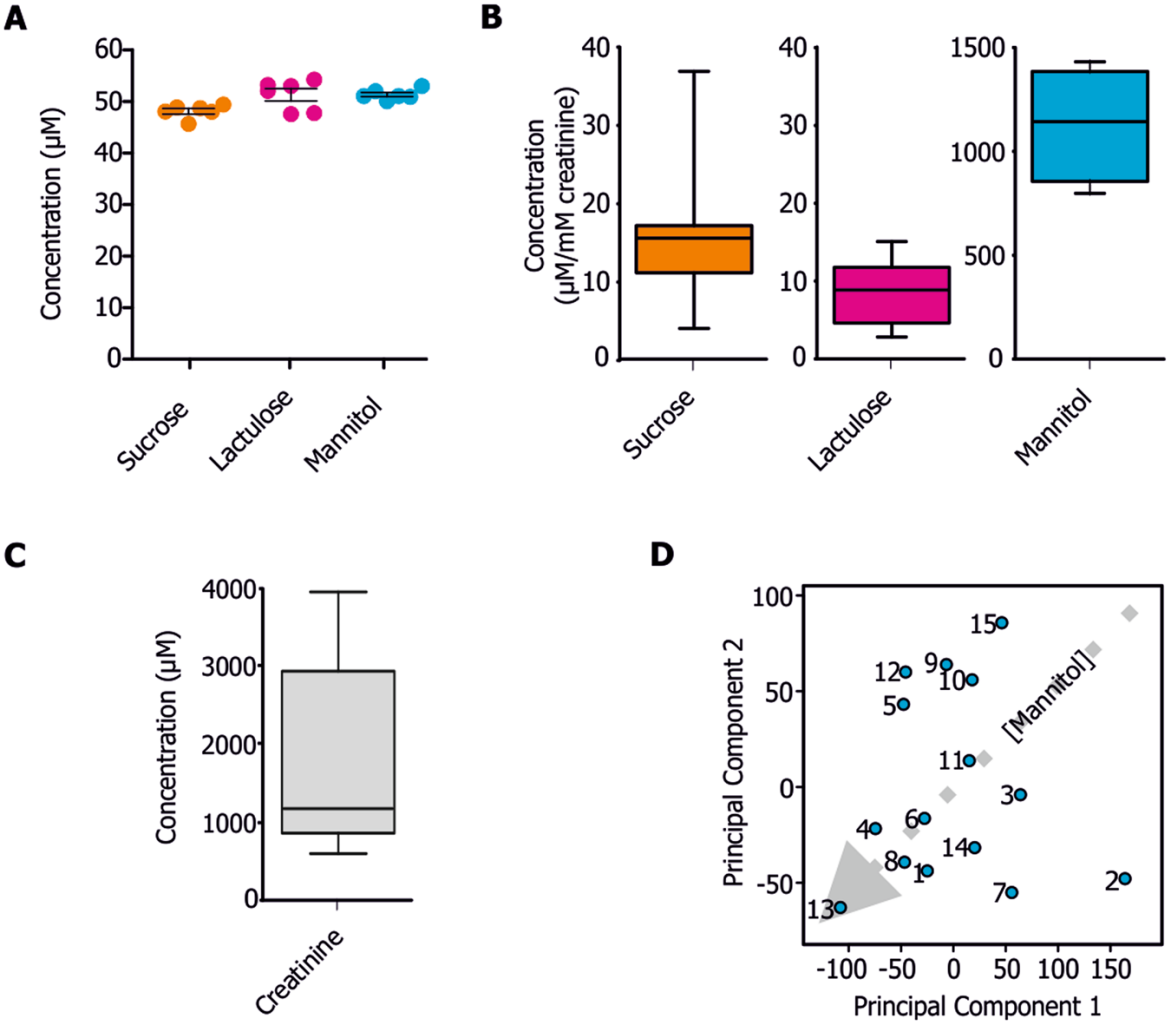
(**A–D**) Quantification of carbohydrates in urine using Chenomx NMR Suite Professional 8.2. (**A**) Spike-in experiments (N = 6) of sucrose, lactulose and mannitol. Error bars represent SD. (**B**) Boxplot representation of carbohydrate concentrations in urine samples. (**C**) Boxplot representation of creatinine concentrations in urine samples. (**D**) Principal component analysis of ^1^H 1D CPMG spectra. Variation explained by principal component 1 (53.7%) and principal component 2 (29.9%).

We first validated our approach using a carbohydrate spike-in experiment. Hereby, defined amounts of carbohydrates (50 µM) were added to urine samples and the amount of sucrose, lactulose and mannitol determined by our NMR-based approach was 48.1 ± 1.3 µM, 52.4 ± 3.7 µM and 54.3 ± 2.6 µM, respectively. Values fluctuating around 50 µM indicate that variations are caused by random errors (i.e. pipetting) and that all signal can be recovered by NMR spectroscopy (Fig. 3A). Subsequently, the amount of excreted sugar was determined in clinical samples. 15 healthy individuals were tested and amount of sugars in urine were detected and quantified (Fig. 3B, Suppl. Table 1). As expected, high variations in sugar concentrations between the individual samples can be observed which are due to the fact that urine is a highly variable biofluid, depending on the individual water and food intake. Similar variations but even higher concentrations of carbohydrates would be expected for the analysis of clinical samples^5,9,10^. Normalization of the carbohydrate concentrations to creatinine levels was performed (Fig. 3C, Suppl. Table 1, Suppl. Fig. 6).

The presented method enables a quick measurement of urine samples and a subsequent manual quantification of carbohydrates. However, for clinical applications with hundreds of samples, a batch processing approach is needed in order to automate not only the process of measurement, but also the following data analysis. Therefore, direct integration of carbohydrate peaks in 2D JRES experiments was performed. Due to large differences in relaxation times (T_1_) of the methyl-groups of the internal standard and creatinine to the carbohydrates of interest (Suppl. Fig. 5), a long relaxation delay of 16 seconds was used for these experiments. Integrals of reference carbohydrate spectra were used in order to calculate concentrations in urine samples. This excludes any quantification differences due to different relaxation times, but also due to any features of JRES experiments (Suppl. Fig. 6, Suppl. Table 2).

As an alternative approach and to obtain a quick readout on carbohydrate concentrations, a principal component analysis was performed and provided hints for high mannitol concentrations within the samples (Fig. 3D). In case of high gastric and/or intestinal permeability, sucrose and lactulose may lead to separation in the principal component analysis.

## Discussion

NMR spectroscopy has, next to mass spectrometry, evolved in the recent years as key technique for the detection and identification of metabolites^17,25–27^. NMR is superior regarding quantification and reproducibility, providing that guidelines for sample preparation and experimental setup are followed^27^. Here we show that NMR spectroscopy is well-suited as urine ‘triple-sugar test’ and enables rapid and robust quantification of lactulose, mannitol and sucrose down to low micro-molar concentrations (Fig. 3B, Suppl. Fig. 7, Suppl. Tables 1 and 2). Although this covers the typical concentration ranges expected for a clinical setting, the limit of detection can be reduced by recording longer NMR experiments.

For research studies with a small set of samples, and if exact quantification of low carbohydrate amounts is of interest, our presented setup is already well suitable. However, in a clinical setting, where hundreds of samples are screened, a faster, automated approach is needed. This can be achieved by (i) decreasing the measurement time and (ii) automated quantification. This is feasible by using our NMR spectroscopy approach, since (i) pathologically and therefore clinically relevant increased intestinal permeability index (lactulose/mannitol ratio >0.07) would presume high concentrations of carbohydrates, which require only short measurement times (<10 minutes) (9, 10) and (ii) a batch integration of 2D J-resolved experiments in NMR analysis software tools (i.e. MestreNova) enable a simultaneous quantification of carbohydrates within several minutes (see Suppl. Fig. 7). Given a mannitol concentration of 1000 µM in a urine sample, a pathologically increased permeability index would presume a lactulose concentration of at least 70 µM within the same sample. With our setup (measurement time 38 min), we can cover carbohydrate concentrations in the low µM range (5 µM), which is ten times lower than the concentration that would indicate an increased permeability in a 1000 µM mannitol sample. By decreasing the number of scans in our setup about factor of 4, we decrease experimental time to <10 minutes and signal-to-noise ratio about a factor of 2, which means that we still detect low concentrated sugars (~10 µM).

This adjusted setup is comparable to a HPLC-based study of Kubica *et al*. in terms of the required measurement time (experimental time: 8 minutes/sample)^11^. The analytical reproducibility and robustness of our NMR-based approach and the much lower interference with other compounds (i.e. sugars) makes it well-suited for clinical applications^28^.

This presented protocol for determination of gastric and/or intestinal permeability is suitable for clinical applications, however, NMR spectroscopy provides additional potential for future implementations. These include e.g. the ERETIC method (Electronic REference To access *In vivo* Concentrations), which provides a reference signal, synthesized by an electronic device and can be used for the determination of absolute concentrations within samples^29–31^.

Summarizing, our approach enables the detection of increased gastric and/or intestinal permeability which is not only a hallmark of liver cirrhosis, but also of celiac disease, Crohn’s disease, type 1 diabetes and food allergies^1^. This fact represents the vital importance of developing a robust and reliable protocol for the detection of increased gastric and intestinal permeability.

## Methods

### Subjects

The cohort study was performed in Graz from December 2016 until March 2017. Inclusion criteria for the study were (I) age of 24 years or above, (2) abstinence from alcohol 48 h prior to study visit and (3) the willingness and ability to give informed consent. Exclusion criteria for the study were (1) Alcohol Use Disorders Identification Test 8 or above, (2) CAGE test 2 or above, (3) elevated liver function tests, (4) pregnancy, (5) breast-feeding and (6) any disorder or medication that does not allow concomitant consumption of alcohol. No diagnostic criteria were included, all participants (11 male, 5 female) were healthy, with an age of 26 ± 4, a body weight of 82.3 ± 15.6 kg and a BMI (kg/m^2^) of 26.4 ± 5.2.

All study procedures were performed after obtaining written informed consent and study was conducted according to the principles of the Declaration of Helsinki. The experimental protocol was approved by the institutional review board of the Medical University of Graz (28–255 ex 15/16) and is registered at clinicaltrials.gov (NCT02568904, 06/10/2015).

### Sample collection

Human urine samples of 15 healthy individuals were collected as a full sample 5 hours after intake of an aqueous solution containing 20 g sucrose, 10 g lactulose and 5 g mannitol^2^. The intake, but not the activity was controlled during these 5 hours. For inhibition of fungal growth, Thimerosal (1 mg/ml) was added to urine samples prior to storage at −80 °C.

### Chemicals

Lactulose (Laevolac) was obtained from Fresenius Kabi Austria, D-Mannitol (Fluka), sucrose and thimerosal were obtained from Sigma Aldrich Austria. Sodium phosphate, dibasic (Na_2_HPO_4_), sodium hydroxide, hydrochloric acid (32% m/v), and sodium azide (NaN_3_) were obtained from VWR International, 3-(trimethylsilyl) propionic acid-2,2,3,3-d4 sodium salt (TSP) from Alfa Aesar (Karlsruhe, Germany), deuterium oxide (^2^H_2_O) from Cambridge Isotope laboratories, Inc. (Tewksbury, MA).

### Sample preparation

Urine samples were mixed with 10% deuterated phosphate buffer (1.5 M Na_2_HPO_4_, 3.236 mM TSP, 0.04 (w/v)% NaN_3_; pH adjusted to 7.4 with 8 M HCl and 5 M NaOH) for NMR measurements and transferred into 5 mm NMR tubes (final volume of 600 µL). Samples were stored at −20 °C until measurement.

### NMR experiments

All NMR experiments were performed at 310 K on a Avance Neo Bruker Ultrashield 600 MHz spectrometer equipped with a TXI probe head with Bruker Topspin version 4.0.2. The cpmgpr1d pulse sequence (Bruker, size of fid 73728, 11904.76 Hz spectral width, 128 scans, d1 = 4 s, D20 of 0.3 ms and L4 of 128) was used for recording ^1^H one dimensional experiments with a T_2_ filter using Carr-Purcell-Meiboom-Gill sequence and f1 presaturation for water signal suppression (experimental time 15 min 1 sec). Using this pulse sequence all non-exchangeable protons of small molecules can be detected in a deuterated buffer solution and signal of proteins is suppressed. For Chenomx quantification, the jresgpprqf pulse sequence (Bruker, size of fid 16384/32, 10000.00/16.6583 Hz spectral width in F2/F1, 4 scans, d1 = 2 s), was used for recording ^1^H homo-nuclear J-resolved 2D correlation experiments with presaturation during the relaxation delay (experimental time 27 min 58 sec). For MestReNova batch processing, the jresgpprqf pulse sequence (Bruker, size of fid 16384/32, 10000.00/16.6583 Hz spectral width in F2/F1, 4 scans, d1 = 16 s), was used for recording ^1^H homo-nuclear J-resolved 2D correlation experiments with presaturation during the relaxation delay (experimental time 37 min 54 sec). Using this pulse sequence all non-exchangeable protons of small molecules can be detected in a deuterated buffer solution.

The noesygppr1d pulse sequence (Bruker, size of fid 98304, 17857.143 Hz spectral width, 128 scans, d1 = 4 s) was used for recording ^1^H one dimensional experiments with presaturation delay during relaxation and mixing time. (experimental time 14 min 56 sec). Using this pulse sequence all non-exchangeable protons can be detected in a deuterated buffer solution.

The reset_psyche_1d pulse program with a pseudo 2D sequence and broadband homo-nuclear decoupling using the psyche element (Bruker, size of fid 4096/1024 F2/F1, 11904.762/11904.762 Hz spectral width in F2/F1, 8 scans, d1 = 16 s) was used for recording decoupled ^1^H one dimensional experiments (experimental time 3 h 50 min 1 sec, or 1 h, 4 min 0 sec). Using this pulse sequence all non-exchangeable protons can be detected in a deuterated buffer solution and complex splitting patterns will be resolved.

The dipsi2gpphpr pulse sequence (Bruker, size of fid 8192/400 F2/F1, 11904.76/19.8313 Hz spectral width in F2/F1, 16 scans, d1 = 2 s and a mixing time (D9) of 0.079999998 s) was used for recording homo-nuclear, phase sensitive HartmanαHahn transfer experiments. (experimental time 2 h 12 min 43 sec). Using this pulse sequence all non-exchangeable protons can be detected in a deuterated buffer solution.

Due to high water content, T_1_ determination experiments were performed with a modified version of the zggpw5 pulse sequence preceding a saturation recovery block (delays 0.01, 0.22, 0.54, 1.05, 1.84, 3.08, 5.01 (duplicated), 8.01, 12.69, 20.00 s) before the first 90° pulse and using water suppression with the w5 sequence (Bruker, size of fid 73728/11 F2/F1, 11904.76/5882.353 Hz spectral width in F2/F1, 128 scans, 0.1 s relaxation delay) (experimental time 3 h 25 min 7 sec).

### Spectral processing

Spectral data were all processed in Bruker Topspin version 4.0.2. One-dimensional exponential window multiplication of the FID, Fourier transformation and phase correction. For DIPSI experiments baseline correction, window multiplication and phase correction was performed. Processing was done using QSINE window function (SSB = 2) in both dimensions with real size of spectrum 1024/1024 F2/F1. Fourier transform was performed with 2048/400 F2/F1 points of fid. 2D J-resolved experiments were processed using Bruker processing AU program proc_jres.be. Hereby a back prediction produces a symmetric echo and as a result, FID and apodization maxima are on the very spot^32–34^. Absorptive positive projections of the processed two-dimensional spectra for Chenomx quantification were calculated. Processing of pseudo two-dimensional reset psyche experiments was performed using Bruker AU program proc_reset.

Saturation recovery experiments for T_1_ determination were processed in the F2 dimension, phase corrected and imported into Bruker Dynamics Center for T_1_ determination.

### PCA

NMR data were imported to Matlab® vR2014a (Mathworks, Natick, Massachusetts, United States), regions around the water and TSP signals excluded, and probabilistic quotient normalization was performed to correct for differences in sample metabolite dilution.

To identify changes in carbohydrate contents, multivariate statistical analysis was performed using MetaboAnalyst (4.0).

### Quantification

Quantification of sugars and creatinine was performed using Chenomx NMR Suite Professional 8.2 (Chenomx Inc.). Projections of the indirect dimension of two-dimensional J-resolved spectra of reference sugars with TSP as internal standard were imported into the Library Manager using the Compound Builder Module. Hereby, concentration of TSP as chemical shape indicator and sugar concentrations were defined within the reference samples. NMR signals of reference compounds in isolated standard samples were selected to provide a unique signature of sugars in Chenomx profiler. For proper peak assignment and integration of signals, the transformation of peak clusters was limited to a defined region. The imported spectra in the Library Manager were used in Chenomx Profiler for integration and quantification. Stated concentrations correspond to final concentrations in urine.

For automation of the approach, 2D quantification was performed. Hereby, processed 2D J-resolved experiments were imported into MestReNova 11.0 and peaks corresponding to carbohydrate/creatinine signals were integrated. Absolute concentrations were calculated based on integrals of external standards with known concentrations (determined in MestReNova 11.0). Stated concentrations correspond to final concentrations in urine.

## Electronic supplementary material

Supplementary Information
