# Supplementary material for: NMR spectroscopy enables simultaneous quantification of carbohydrates for diagnosis of intestinal and gastric permeability
Source: Sci Rep. 2018 Oct 2;8:14650. doi: 10.1038/s41598-018-33104-8 (PMC6168465; doi:10.1038/s41598-018-33104-8)
Supplement: Supplementary file 1 — Supplementary Information [file 41598_2018_33104_MOESM1_ESM.pdf]

NMR spectroscopy enables simultaneous quantification of carbohydrates for diagnosis of intestinal and gastric permeability

Detection of gastrointestinal permeability by NMR

Sarah Stryeck[1], Angela Horvath[2,3], Bettina Leber[4], Vanessa Stadlbauer[2], Tobias Madl[1*]

Supplementary Figures

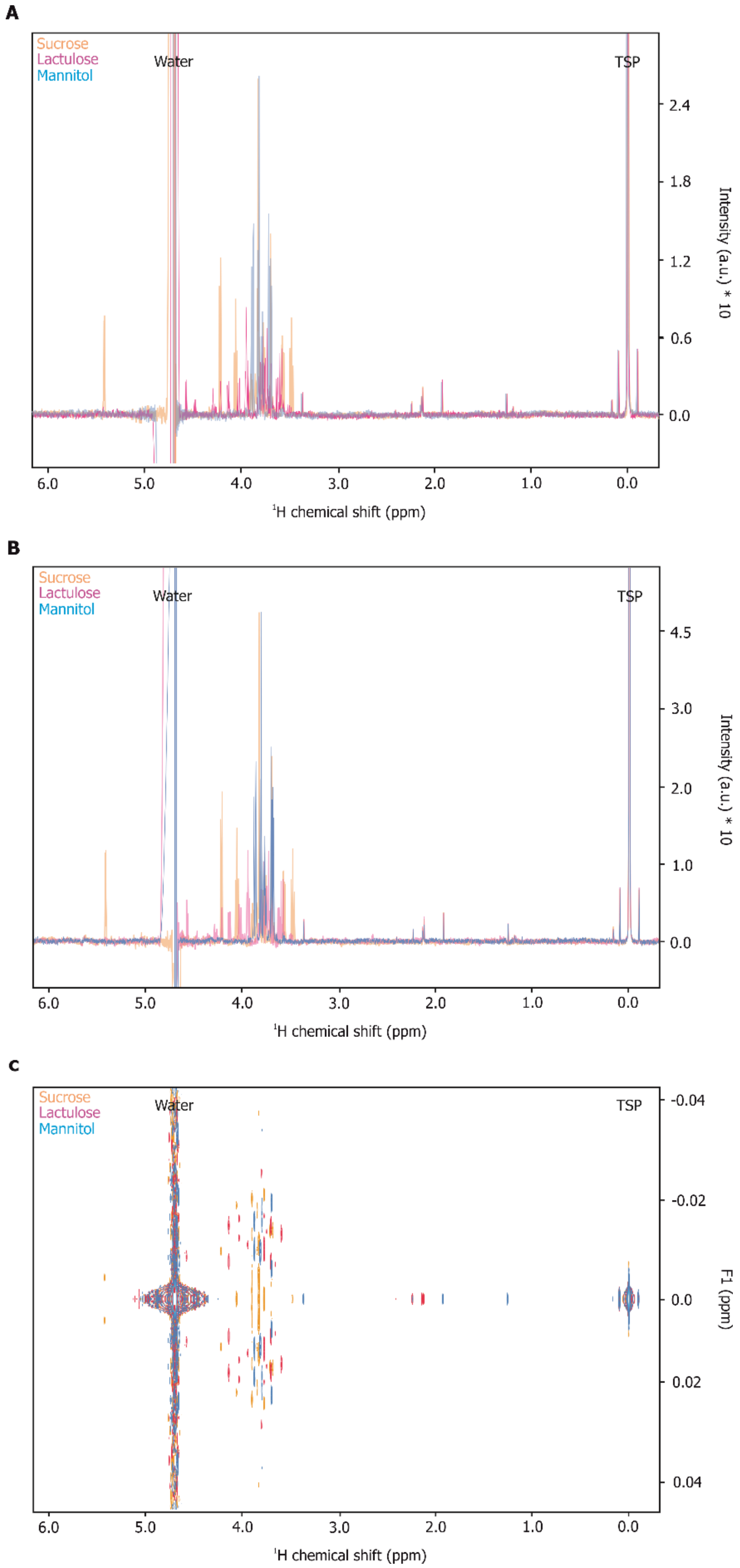

Suppl. Fig. 1 (A-C): Reference NMR spectra of carbohydrate standards (full spectrum). A. Overlay of ^{1}H 1D CPMG experiments of sucrose (orange), lactulose (magenta) and mannitol (light blue) (each 100 µM). B. Overlay of ^{1}H 1D NOESY experiments of sucrose (orange), lactulose (magenta) and mannitol (light blue) (each 100 µM). C. Overlay of ^{1}H 2D J-resolved experiments of sucrose (orange), lactulose (magenta) and mannitol (light blue). (each 100 µM)

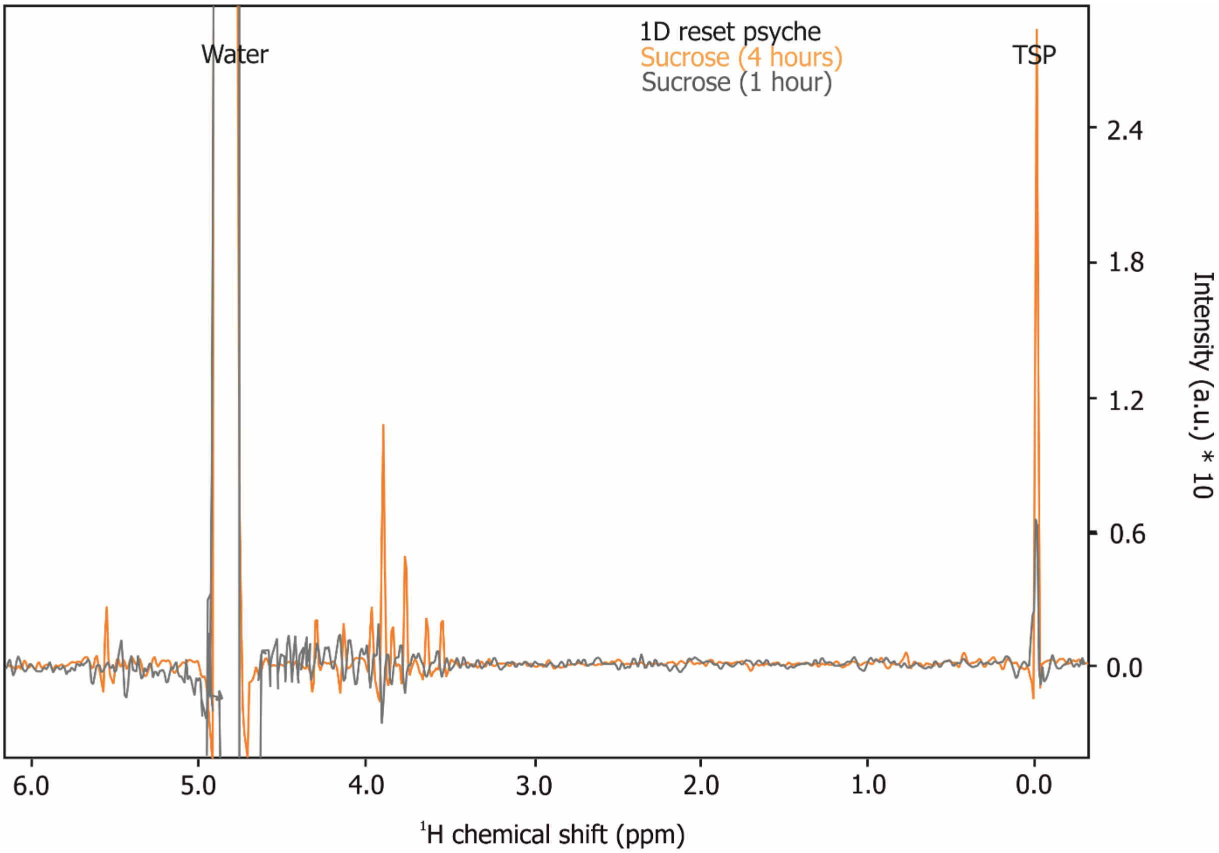

Suppl. Figure 2: Homo-nuclear decoupling. Overlay of homo-nuclear decoupled psyche experiment with one hour (grey) and four hours (orange) measurement time of a 1 mM sucrose standard.

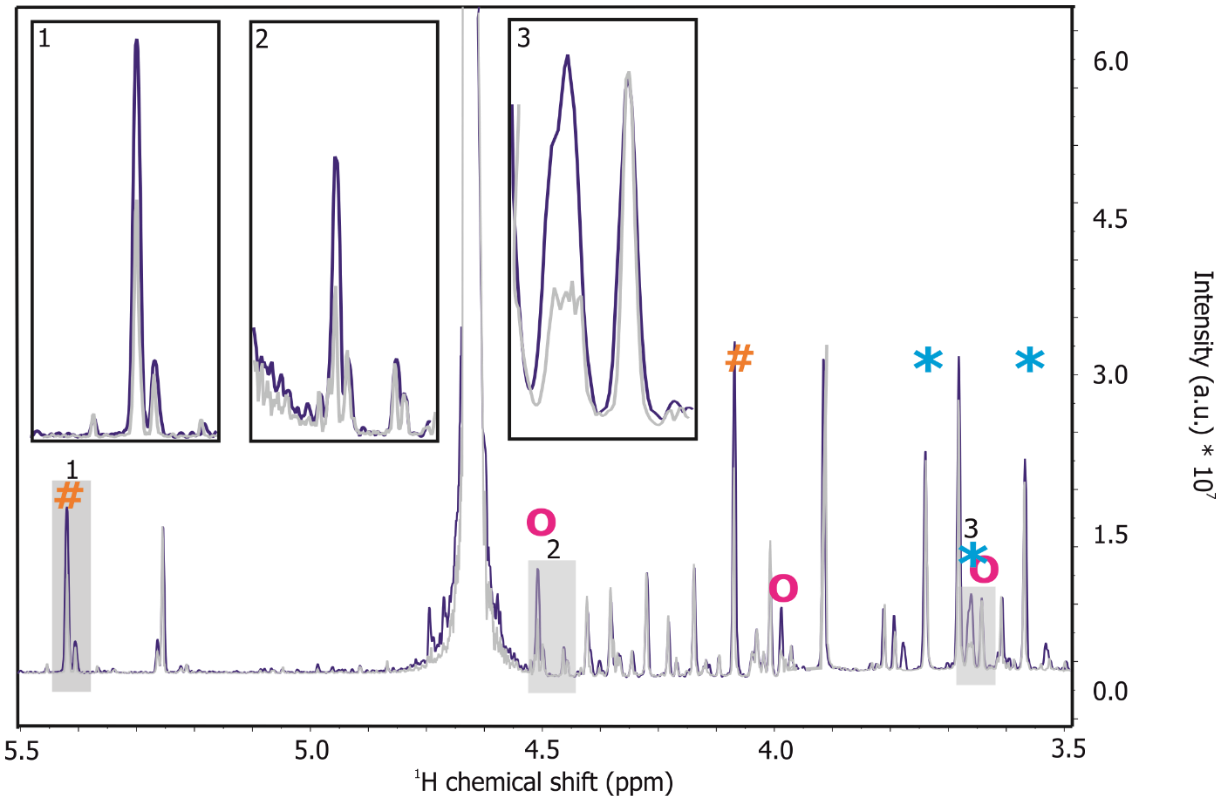

Suppl. Figure 3: NMR spectroscopy of urine samples. Projections of ^{1}H J-resolved 2D experiments of reference compounds sucrose (orange), lactulose (magenta) and mannitol (light blue) (each 100 µM). Corresponding resonances of these sugars in a human urine sample after sugar intake (grey) and increased signal after spike-in (marine blue) are indicated with orange hash (sucrose), magenta circle (lactulose) or light blue asterisks (mannitol).

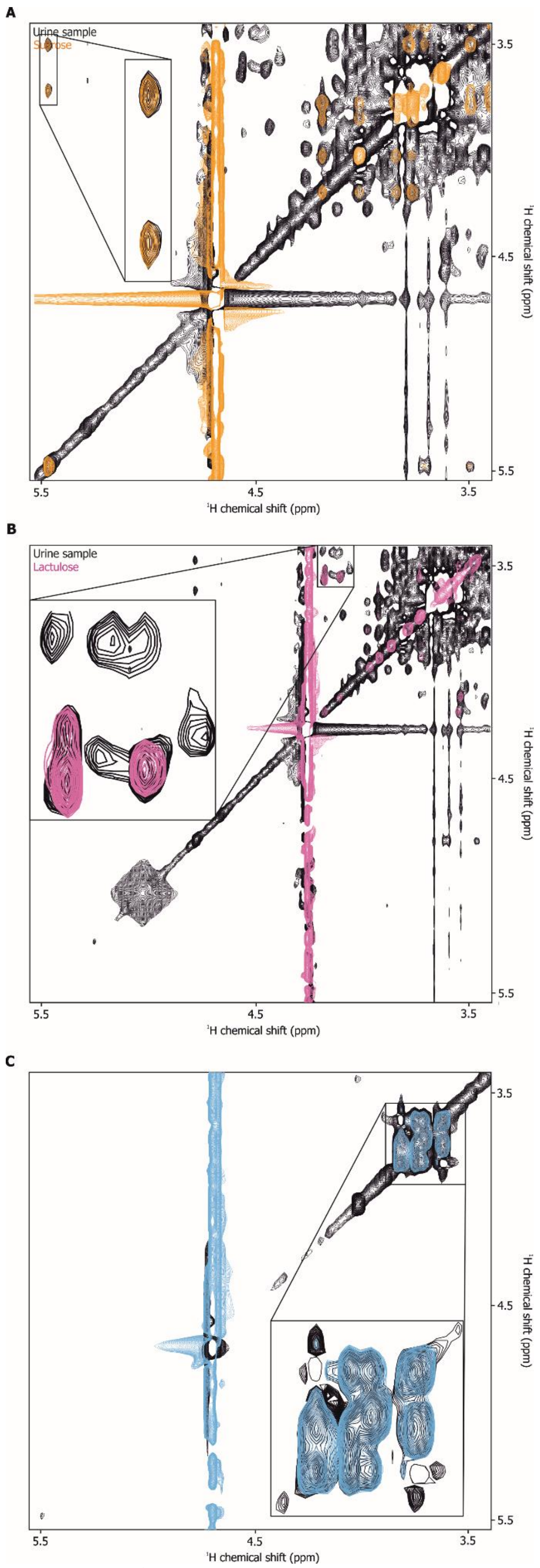

Suppl. Figure 4 (A-C): Assignment of carbohydrates in a sugar sample. A. Overlay of 2D ^{1}H DIPSI experiment of a human urine sample (black) with sucrose standard (orange). B. Overlay of 2D ^{1}H DIPSI experiment of a human urine sample (black) with lactulose standard (magenta). C. Overlay of 2D ^{1}H DIPSI experiment of a human urine sample (black) with mannitol standard (light blue).

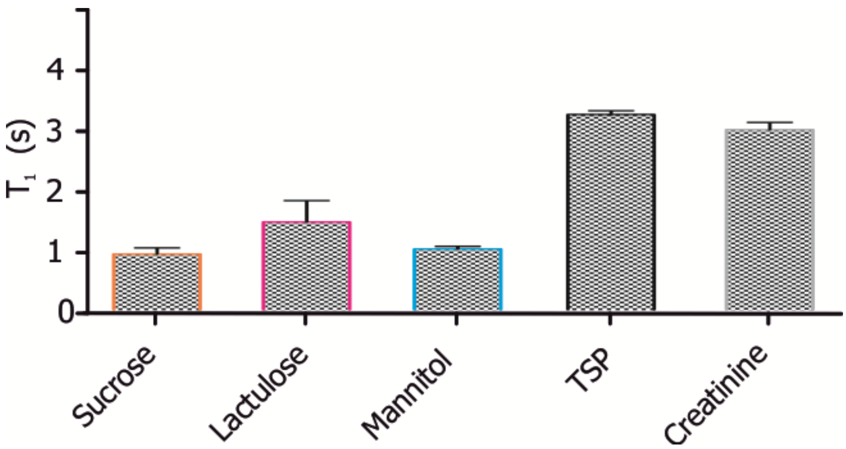

Suppl. Figure 5: T_1 times for studied compounds. Bar diagram indicating T_1 times for carbohydrates, TSP and creatinine.

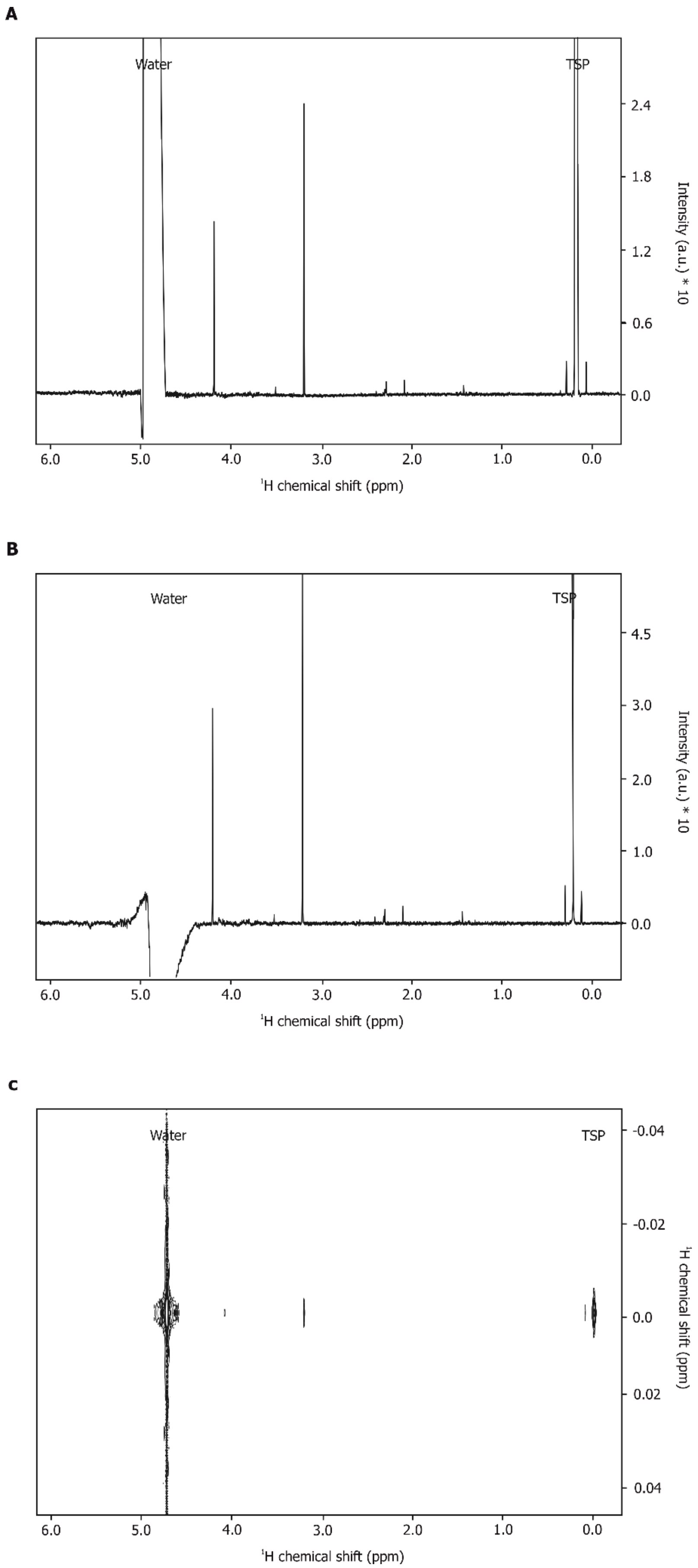

Suppl. Figure 6 (A-C): Reference NMR spectra of creatinine standard (100 µM). A. ^{1}H 1D CPMG experiment of creatinine. B. ^{1}H 1D NOESY experiment of creatinine. C. ^{1}H 2D J-resolved experiment of creatinine.

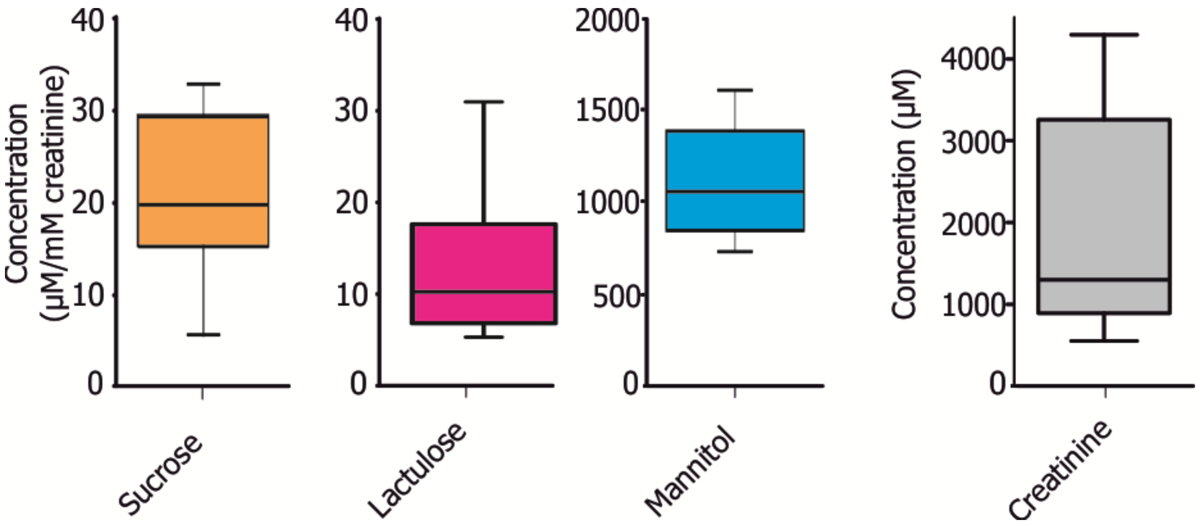

Suppl. Figure 7: Quantification of carbohydrates in urine using 2D J-resolved experiments. Boxplot representation of carbohydrate concentrations [µM/mM creatinine] in urine samples and µM concentration of creatinine.

Supplementary Tables

Supplementary Table 1: Concentrations of sugars in human urine samples after sugar intake in µM/mM creatinine determined by Chenomx quantification.

ID	Mannitol	Sucrose	Lactulose
**Sample 1**	1136.7	11.1	8.5
**Sample 2**	847.6	n.d.	n.d.
**Sample 3**	794.0	n.d.	n.d.
**Sample 4**	1342.6	36.8	15.0
**Sample 5**	937.3	15.5	2.8
**Sample 6**	1376.6	12.1	n.d.
**Sample 7**	1422.9	23.6	9.1
**Sample 8**	1419.6	15.9	10.6
**Sample 9**	851.5	n.d.	n.d.
**Sample 10**	980.7	n.d.	n.d.
**Sample 11**	1001.3	11.8	n.d.
**Sample 12**	836.2	5.9	5.2
**Sample 13**	1371.3	4.0	n.d.
**Sample 14**	1394.4	n.d.	n.d.
**Sample 15**	1159.3	16.0	n.d.

Supplementary Table 2: Concentrations of sugars in human urine samples after sugar intake in µM/mM creatinine determined by 2D JRES quantification.

ID	Mannitol	Sucrose	Lactulose
**Sample 1**	887.0	21.0	n.d.
**Sample 2**	759.0	n.d.	n.d.
**Sample 3**	723.0	n.d.	n.d.
**Sample 4**	1198.0	32.0	12.0
**Sample 5**	828.0	31.0	n.d.
**Sample 6**	1440.0	n.d.	n.d.
**Sample 7**	1253.0	20.0	n.d.
**Sample 8**	1475.0	16.0	13.0
**Sample 9**	1003.0	n.d.	31.0
**Sample 10**	821.0	n.d.	n.d.
**Sample 11**	999.0	n.d.	5.0
**Sample 12**	756.0	5.0	n.d.
**Sample 13**	1286.0	n.d.	8.0
**Sample 14**	1314.0	18.0	7.0
**Sample 15**	1055.0	14.0	n.d.
